# Dark field nanoparticle tracking analysis for size characterization of plasmonic and non-plasmonic particles

**DOI:** 10.1007/s11051-014-2419-x

**Published:** 2014-05-01

**Authors:** Thorsten Wagner, Hans-Gerd Lipinski, Martin Wiemann

**Affiliations:** 1 Biomedical Imaging Group, Dortmund University of Applied Sciences and Arts, Emil-Figge-Straße 42, 44227 Dortmund, Germany; 2 IBE R&D Institute for Lung Health gGmbH, Mendelstraße 11, 48149 Münster, Germany

**Keywords:** Nanoparticle tracking analysis, Dark field microscopy, Instrumentation development, Surface plasmon resonance, Particle characterization, ImageJ

## Abstract

Dark field microscopy is a widely unknown method to measure the particle size distribution of diffusing nanoparticles by particle tracking. Here we demonstrate that by using the surface plasmonic resonance of Au nanoparticles, size differences of ca. 20 nm can be identified within the particle size distribution. For that purpose, we developed a software tool which helps to analyze color videos of diffusing nanoparticles retrieved from CCD or CMOS cameras. Polystyrene beads with a diameter of 100 and 200 nm were used to compare the results to those obtained with a well-established laser-based particle tracking system. The methodology will be discussed in the light of recent developments in the emerging field of optical nanoparticle tracking.

**Electronic supplementary material:**

The online version of this article (doi:10.1007/s11051-014-2419-x) contains supplementary material, which is available to authorized users.

## Introduction

The use of gold and silver nanoparticles (NPs) has received much attention in recent years because of their unique light scattering and absorption characteristics (Ringe et al. 2013; El-Brolossy et al. 2008; Kelly et al. 2003; Link and El-Sayed 1999). Due to their plasmonic resonance and the lack of photobleaching or blinking (Cai et al. 2008), these metal particles are used as bio-nano-sensors (Anker et al. 2008). As early as 1902 Siedentopf and Zsigmondy (1902) showed that diffusing Au NPs are visible with dark field microscopy (DFM). They also observed diffraction patterns of NP in multiple colors. This phenomenon, based on the surface plasmonic resonance (SPR) of metal NPs, is influenced by the dielectric constant, the temperature of the surrounding medium, and mainly by particle size and shape (Link and El-Sayed 1999; Kelly et al. 2003; Buecker 2007; El-Brolossy et al. 2008; Bingham and Willets 2009). Thus, under ideal conditions, the color information offers the opportunity to characterize the size of NPs with refined methods. Since a couple of years a size measurement technique called nanoparticle tracking analysis (NTA) is increasingly used to determine the size distribution of NPs in liquids. This NTA technique makes use of intense laser light to illuminate freely diffusing particles, tracks their Brownian motion by analysis of monochrome images, and finally, estimates their size distribution using the Stokes–Einstein relation. As the technique is applicable to biological fluids and to comparatively low particle concentrations, it has become major importance e.g., for measuring NPs in the field of nanotoxicology (Buzea et al. 2007; Yang et al. 2010; Stone et al. 2010). Another widely used method for particle characterisation is dynamic light scattering. This technique determines the size distribution of a nanoparticle collective (down to 1 nm) and is well suited for concentrated nanoparticle suspensions, whereas it is disturbed by larger, gravitationally settling particles. The latter is not a problem if single particles are tracked by NTA (Filipe et al. 2010; Hole et al. 2013) or the tracking method used in this paper. This method takes advantage from DFM, which has previously been applied to other scientific issues. Sönnichsen and Alivisatos (2005) used single particle tracking to estimate the plasmon-based orientation of Au nanorods; Bingham and Willets (2009) tracked single Ag NPs, analyzed their scattering spectra, and estimated their diffusion coefficient simultaneously; and Sagle et al. (2012) tracked single Au NPs for the study of bilayers with ganglioside lipids to distinguish between random and confined diffusion. However, little attention has been paid to the capabilities of DFM to estimate size distribution of suspended NP. This paper, therefore, focuses on the advantages and limitations of calculating size distributions of NP from DFM videos by means of nanoparticle tracking. In particular, we will demonstrate that the color information of NP retrieved from CCD or CMOS cameras bears valuable information which may help identify subpopulations of metal NP. Therefore, an ImageJ-based software called NanoTrackJ (Wagner et al. 2014) was developed which is capable to analyze a wide range of particle videos including their color information.

## Material & methods

### Particles

Gold NPs (BBI, Cardiff, UK), nominally sized 60 and 80 nm, were used due to their stability and pronounced SPR. These particle qualities were diluted in essentially particle-free double distilled H$_{2}$O and mixed to obtain suitable bimodal particle suspensions. For verification purposes, 100 nm (Kisker-Biotech, PPs-0.1) and 200 nm (Kisker-Biotech, PPs-0.2) polystyrene particles were used. Trackable concentrations amounted to approximately $5\times 10^{8}/\text {ml}$ and $3\times 10^{9}/\text {ml}$ for NTA and dark field measurements, respectively.

### Imaging equipment and data acquisition

Image data were collected with a NanoSight^TM^ LM10 system equipped with a LM14 green (535 nm) laser module and a cooled Andor camera (Andor-DL-658-OEM). For DFM, an Olympus BX51 microscope was used which was illuminated with a CytoViva^TM^ dark field oil condenser. Particle suspension was pipetted into a micro-chamber consisting of a cleaned slide and a cover slip which was supported by two cover slip fragments, thus defining the height of the chamber to ca. $160\,\upmu \text {m}$. A 100-fold oil immersion objective with iris aperture (Olympus) was brought in place and the optimal dark field illumination was adjusted with a completely closed iris. Measurements were carried out at a defined temperature ($22- 24\, ^{\circ }\text {C}$). Color image series were taken with a digital single-lens reflex camera (Canon EOS 5D Mark II) set to the highest sensitivity (ISO 3200) in the movie mode (25 fps, resolution 1920 $\times$ 1020 px) or with a PCO Pixelfly Edge camera (PCO AG, Kehlheim, Germany, black and white custom model). The NTA measurements were analyzed by the NTA 2.3 software and by the newly developed NanoTrackJ software. Dark field measurements were analyzed by NanoTrackJ only. All captured video files and settings to reproduce the measurements are listed in the supplementary information.

### Nanoparticle tracking analysis

NTA adapts some principles of single particle tracking to estimate size distributions of NPs in liquid suspensions. To estimate a size distribution, four steps are necessary: (i) Each particle in each frame has to be identified and its center needs to be calculated, (ii) each identified particle has to be tracked for an acceptable time period, (iii) the diffusion coefficients have to be estimated by means of the particle trajectories, and (iv) the size distribution is calculated considering temperature and viscosity. The following section describes how these issues are solved by NanoTrackJ, and gives advice as to which method provides most robust results.

#### (i) Identifying each particle

The objective of this step is to segment each diffraction pattern and estimate the center of it. NanoTrackJ offers three alternative ways to solve that problem: (i) utilize the “Find Maximum” method, (ii) combine the “Find Maximum” method and a Gaussian fit procedure, and (iii) let the user segment the video data on his own and use the binary video data.

Method (i) was used throughout all measurements conducted with NanoTrackJ and works as follows: The 8-neighborhood of each pixel is scanned for higher pixels. If no higher pixel is found, the center pixel is marked as the maximum; all maximum values are sorted by intensity value in descending order, and commencing with the first local maximum (in sorted order) a flood filling algorithm is carried out. This flood filling algorithm groups all pixels around the respective maximum value and uses a pre-set intensity tolerance. If another local maximum is located inside this grouped region, it will be discarded. The centers of gravity of the grouped regions are returned as maximum values.

Method (ii) provides center estimations with “sub-pixel accuracy.” Starting from the position returned by method (i), it estimates the spread of the main maximum of the diffraction pattern. Subsequently, it fits a 2D Gaussian distribution to the main maximum of the diffraction pattern. The center of this Gaussian fit, theoretically, provides subpixel accuracy (Cheezum et al. 2001). However, the main maximum often suffers from saturation effects or lacks a Gaussian shape, such that a subpixel accuracy cannot be reached in these cases.

Method (iii) works with binary image sequences only. It is useful for data that could not be analyzed by method (i) or (ii). The user has to segment the footage using ImageJ before the image sequence can be analyzed by NanoTrackJ. Then, the center of gravity of each connected component is calculated.

#### (ii) Tracking each particle

After identifying each particle in every frame, the particle positions have to be connected to a trajectory (or “track”). For this purpose, the software has to track the particle in the image sequence. In NanoTrackJ, this is done as follows: Let an image sequence consist of N frames $F=\{F_{\triangle t},F_{2\triangle t},\ldots ,F_{N\triangle t}\}$ captured at a constant time interval of $\triangle t$. Let $K_{i\triangle t}$ be the set of identified particles in frame $F_{i\triangle t}$ and $1\le i\le N$. Given a particle with a position $p_{j}$ with $0<j<|K_{i\triangle t}|$ in frame $F_{i\triangle t}$ then $M_{j}$ is the set of particles in the previous frame $F_{(i-1)\triangle t}$ which are inside a circle with the radius r centered at the position $p_{j}$:

1 $$\begin{aligned} M_{j}=\{q:q\in K_{(i-1)\triangle t},\Vert p_{j}-q\Vert <r\} \end{aligned},$$

where $||\cdot ||$ is the euclidean norm. However, the tracking algorithm interconnects the particle positions only if the following conditions are fulfilled:

2 $$\begin{aligned} |M_{j}|&= 1\end{aligned}$$

3 $$\begin{aligned} M_{j}\cap M_{k}&= \{\emptyset \}\quad {\rm for}\, j\ne k \end{aligned}$$

This means that the decision of connecting two particles has to be unique. Eq. (2) ensures that a particle is not connected to more than one particle in the previous frame and Eq. (3) ensures that two particles are not connected to the same particle in the previous frame. If two particles in subsequent frames are connected successfully, it is assumed that these two centers represent the same particle which had changed its positions during $\triangle t$. This change in the particle position is referred to as a “step.” A trajectory of a particle is formed of L steps, where L is proportional to the diffusion time of the particle and is referred to as “tracklength.” In an ideal case, the tracklength covers the complete registration period. However, this occurs rather infrequently because (i) the particle diffuses out of the field of view or (ii) an assignment does not satisfy Eq. (2) or (3). Whereas the first issue depends in part on the optical and instrumental settings (illumination intensity, numerical aperture of the objective, camera sensitivity), the second issue may be minimized not only by preparing samples with a reasonably adapted particle concentration (see "Particles" section ) but also by software. For this purpose, NanoTrackJ is parametrized by a minimum expected particle diameter. Knowing the sample’s temperature and viscosity, the corresponding maximum-expected diffusion coefficient $D_{max}$ can be calculated by the Stokes–Einstein relation. Using this maximum-expecting diffusion coefficient, the search radius r is calculated by

4 $$\begin{aligned} r=3\sqrt{\pi D_{max}\triangle t} \end{aligned},$$

where $\sqrt{\pi D_{max}\triangle t}$ is the mean step length of a particle with the diffusion coefficient $D_{max}$(der Meeren et al. 2012). This radius ensures that more than 99 % of the expected particle steps are shorter than this radius (Wieser and Schütz 2008), provided that there is no smaller particle as specified by the minimum expected particle diameter. It is worthwhile to note that the search radius is of critical importance. A too large radius will result in shorter track lengths, because Eqs. (2) and (3) will be satisfied less often. In case the radius r is too small, steps exceeding r will not become detected, which leads to a smaller mean step length and a bias towards a larger mean particle size.

#### (iii) Diffusion coefficient estimation

Given a particle trajectory, its diffusion coefficient could be calculated in several ways. Two methods are available in NanoTrackJ: The “regression method” and the “covariance method.”

The regression method is the most often used method in the literature to estimate the diffusion coefficient. It evaluates the mean squared displacement $\left\langle d^{2}\right\rangle$ of particle diffusion in $n$ dimensions for different time lags $\tau _{k}=k\triangle t$:

5 $$\begin{aligned} \left\langle d^{2}\right\rangle _{k}=2nD\tau _{k} \end{aligned}$$

Evaluation of Eq. (5) for different $\tau _{k}$ leads to a bunch of data points $(\left\langle d^{2}\right\rangle _{k},k)$. The diffusion coefficient is then estimated by the slope of a regression line fitting a specific number of data points. The slope of this regression line is proportional by $2n\triangle t$ to the diffusion coefficient. This method is very simple but unfortunately error prone. Up to now it’s not clear, how many data points lead to the best estimate. Wieser and Schütz (2008) states that only the first two time lags $(k=1,2)$ should be used and Vestergaard (2012) concluded that the more data points are included in the fit the greater is the error in the estimate. However, Ernst and Köhler (2013) recommend to use the data points with the time lag $k=2$ to $k=5$, and Michalet and Berglund (2012) used an iterative approach to estimate the optimal number of data points. Due to these contradictory recommendations for the correct number of data points, NanoTrackJ allows the user to determine what minimum and maximum time lag should be used.

However, here we decided to use the covariance estimator throughout the paper. Because it is unbiased and needs no parameters, it outperforms the regression method for most diffusion-to-noise ratios and it almost attains the minimum standard deviation for an unbiased estimator, the Cramer–Rao bound (see Appendix). Given the measured position $x[n]$ at discrete timepoints $t_n=n\triangle t$ of a particle diffusing in one dimension, then the covariance estimator is defined in the following way:

6 $$\begin{aligned} \hat{D}=\frac{\overline{\triangle x_{n}\triangle x_{n}}}{2\triangle t}+\frac{\overline{\triangle x_{n}\triangle x_{n+1}}}{\triangle t} \end{aligned},$$

where the second term in the right-hand side is an estimator for the localization noise and

7 $$\begin{aligned} \triangle x_{n}=x[n]-x[n-1] \end{aligned}$$

Please refer to Appendix for a more comprehensive precision analysis of both estimators.

#### (iv) Estimation of the size distribution

Once the diffusion coefficients are estimated, the derivation of a size distribution is straight forward. NanoTrackJ provides two methods for this purpose. The first method simply transforms the diffusion coefficients to a hydrodynamic diameter using the Stokes-Einstein relation and weights each diameter by the track length from which it is derived. Then, a histogram is formed using the weighted diameter data. The weighting is important not only because longer tracks provide more precise estimate but also because smaller particles diffuse in and out the field of view more rapidly and, therefore, more often than larger particles. Consequently, it makes sense to treat each particle step as a single event instead of the complete track (ASTM 2012).

The second method also accounts for this fact by using a maximum likelihood method as suggested by Walker (2012). He defines the following iterative algorithm to estimate the particle size histogram with M radius bins and a bin size of $\triangle r$

8 $$\begin{aligned} P_{m}^{(j+1)}=P_{m}^{(j)}\frac{1}{N}\sum \limits _{n-1}^{N}\left\{ \frac{P_{d}(\triangle _{n};k_{n},m\cdot \triangle r)}{\sum \nolimits _{l=1}^{M}\frac{P_{d}(\triangle _{n};k_{n},l\cdot \triangle r)}{\sum \nolimits _{i=1}^{M}P_{i}^{(j)}}}\right\} \end{aligned},$$

where

9 $$\begin{aligned} P_{d}(\triangle _{n};k_{n},r)=\frac{k_{n}(k_{n}\triangle _{n})^{k_{n}-1}\cdot exp(-k_{n}\triangle _{n}/\theta _{r})}{\theta _{r}^{k_{n}}\cdot \varGamma (k_{n})} \end{aligned}$$

and $\theta _{r}=(2K_{b}T\triangle t)/(3\pi \eta r), \triangle _{n}$ is the mean squared displacement of track $n$ , and $k_{n}$ is the number of steps of track $n$. The algorithm starts from uniform distribution ($P_{m}^{0}$) and updates the bin probabilities for all bins $1\le m\le M$ in one iteration. The mean squared displacement is derived from the estimator chosen in step (iii) by using the relation $\triangle _{n}=2D_{n}\triangle t$. Here the covariance estimator was used, since it is an efficient estimator for both the diffusion coefficient and $\triangle _{n}$ which linearly depends on the former. The algorithm stops, if the differences between the histogram of the displacement data and the maximum likelihood histogram for the displacement are no longer changing. This method leads to very “clean” histograms within which the modal values are easily identified. Throughout the paper, this method was used for all measurements with NanoTrackJ.

## Results and discussion

In the first section, we will demonstrate the ability of NanoTrackJ to determine the correct particle size, using the proprietary NanoSight^TM^ system as a NTA reference system. Then, the utilization of SPR of Au NP for an advanced size measurement will be demonstrated. Finally, advantages and disadvantages of either method will be discussed.

### Quality control of NanoTrackJ

**Fig. 1:**
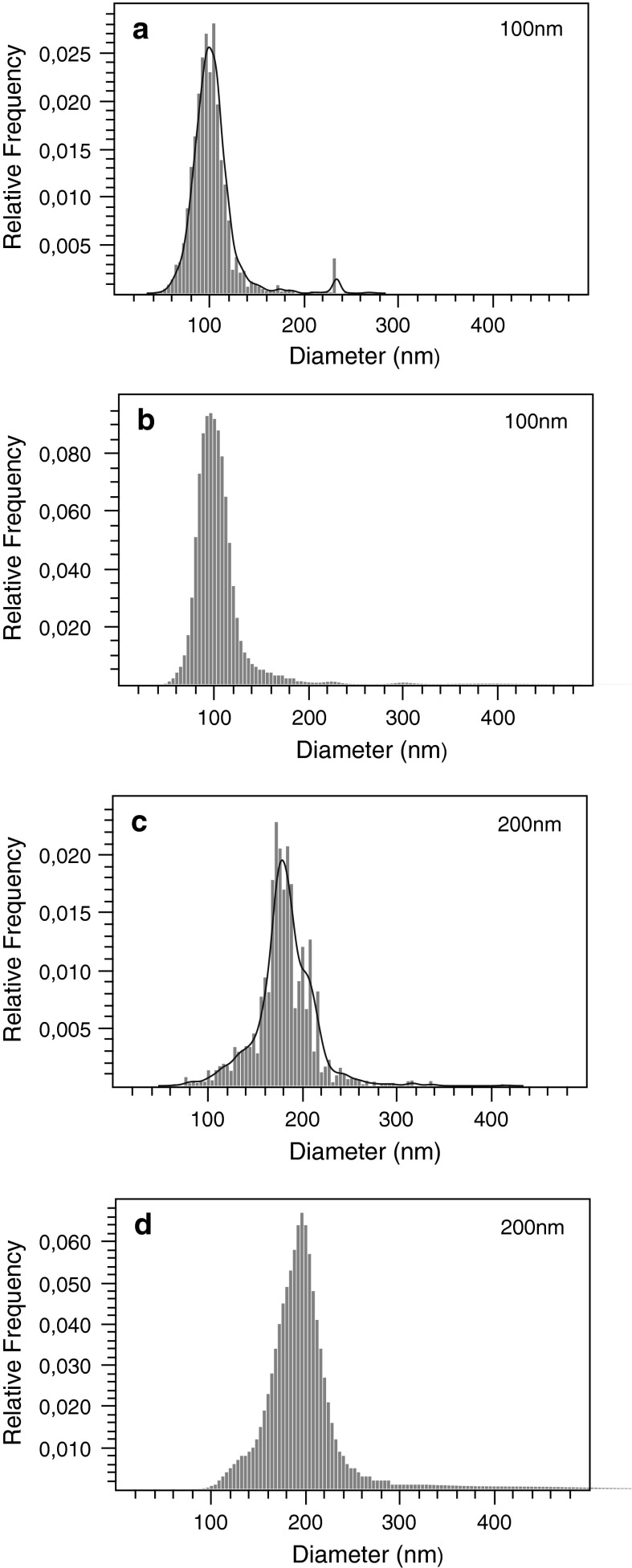
Comparison of NanoTrackJ and NTA using a 100 nm and a 200 nm NP suspension. Video sequences were captured with a NanoSight^TM^ LM10 microscope and analyzed by NTA 2.3 (**a**, **c**) and NanoTrackJ (**b**, **d**). Because NTA 2.3 reports continuous size distributions, we added a kernel density estimate to plot **a** and **c**

**Fig. 2:**
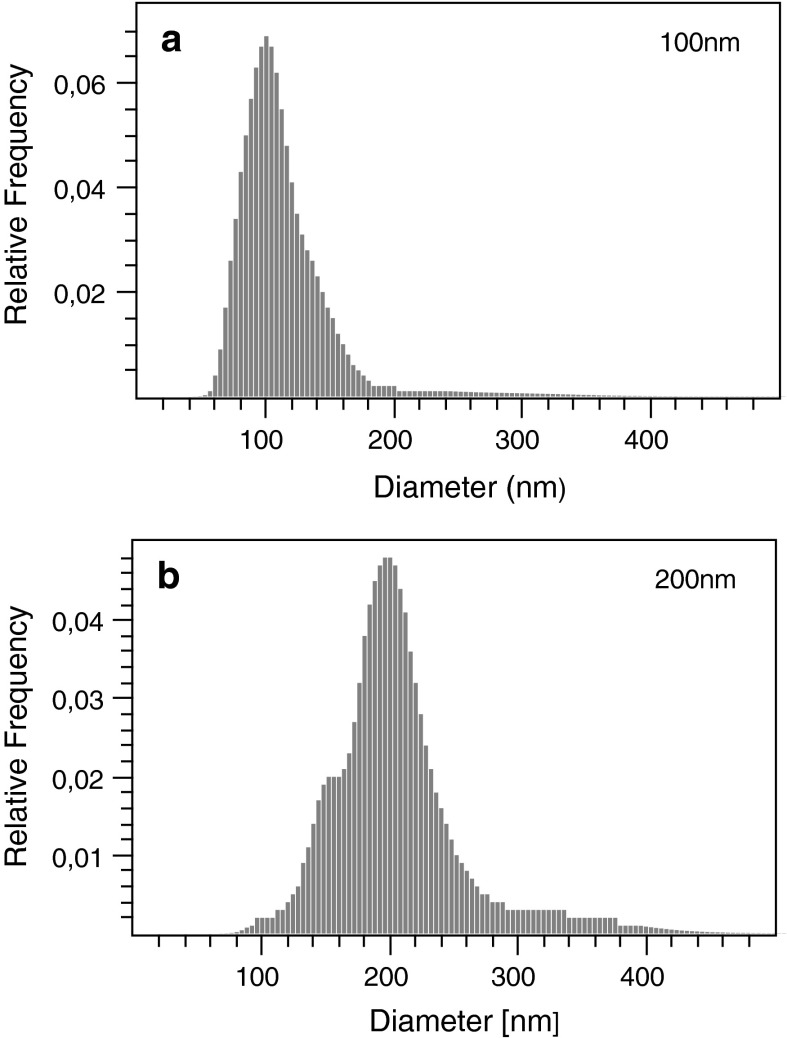
Size distribution of polystyrene NP (100 and 200 nm) viewed with DFM and analyzed with NanoTrackJ. Both histograms were calculated by Walker’s method based on 937 tracks (**a**) / 438 tracks (**b**) with a mean track length of 35 (**a**) / 71 (**b**)

Videos of polystyrene beads diffusing in H$_{2}$O were captured using the NTA device and analyzed by both, the NanoSight^TM^ NTA 2.3 software and NanoTrackJ. Size distributions determined by NanoTrackJ (Fig. 1b, d) agreed well with distributions reported by NTA software (Fig. 1a, c). Data in Table 1 show that not only the modal values match fairly well but also the inter-quantile ranges are in good agreement. Using the same video material, the modal diameter reported for the 200 nm polystyrene video by NanoTrackJ came slightly close to the nominal particle diameter compared to NTA, an effect which may be caused e.g., by the different diffusion coefficient estimators. Although DFM is predestined for analyzing plasmonic NPs (see "Utilizing the surface plasmon resonance for dark field NTA" section), NPs made from polystyrene can be evaluated as well. Figure 2 shows the size distributions of 100 and 200 nm NP viewed with DFM and analyzed with NanoTrackJ. Modal values are centered at the nominal size of the beads indicating the correctness of the method.

**Table 1:** Quantitative validation of NanoTrackJ (NTJ) using the polystyrene experiments shown in Fig. 1

Polystyrene diameter	100 nm	100 nm	200 nm	200 nm
Software	NTA	NTJ	NTA	NTJ
Analysis time (s)	60	60	60	60
Modal value (nm)	101	98	181	196
$IQR_{75-25}$ (nm)	22	24	31	31
#Tracks	1,263	1,290	524	469
Tracklength	53	64	80	109
Figure	1a	1b	1c	1d

**Fig. 3:**
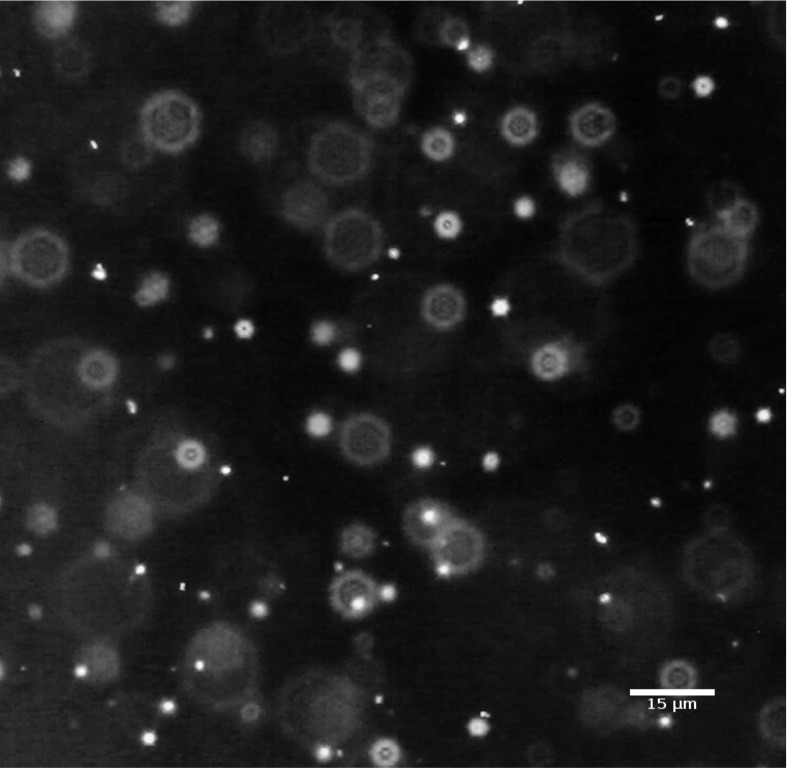
Footage of the color video of a bimodal mixture of 60 and 80 nm Au NPs. A diverging SPR can be seen with dominating colors *green* and *orange*. (Color figure online)

**Fig. 4:**
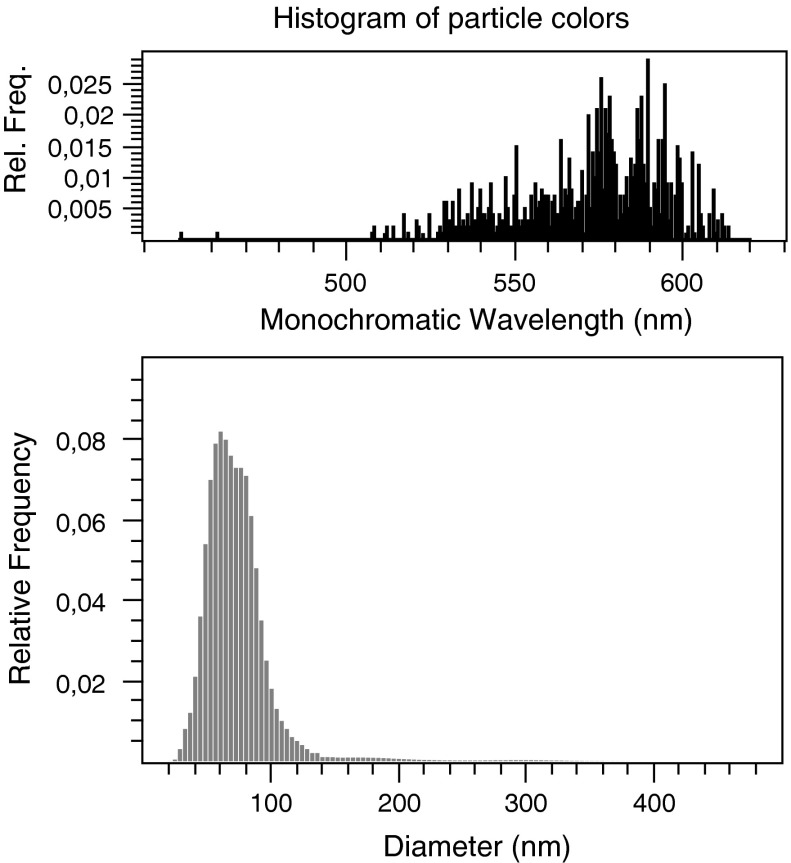
Size distribution of the bimodal AuNP mixture from NanoTrackJ. The 561 tracks of Au NPs shown in Fig. 3 are evaluated. The modal value is 60 nm

### Utilizing the surface plasmon resonance for dark field NTA

**Fig. 5:**
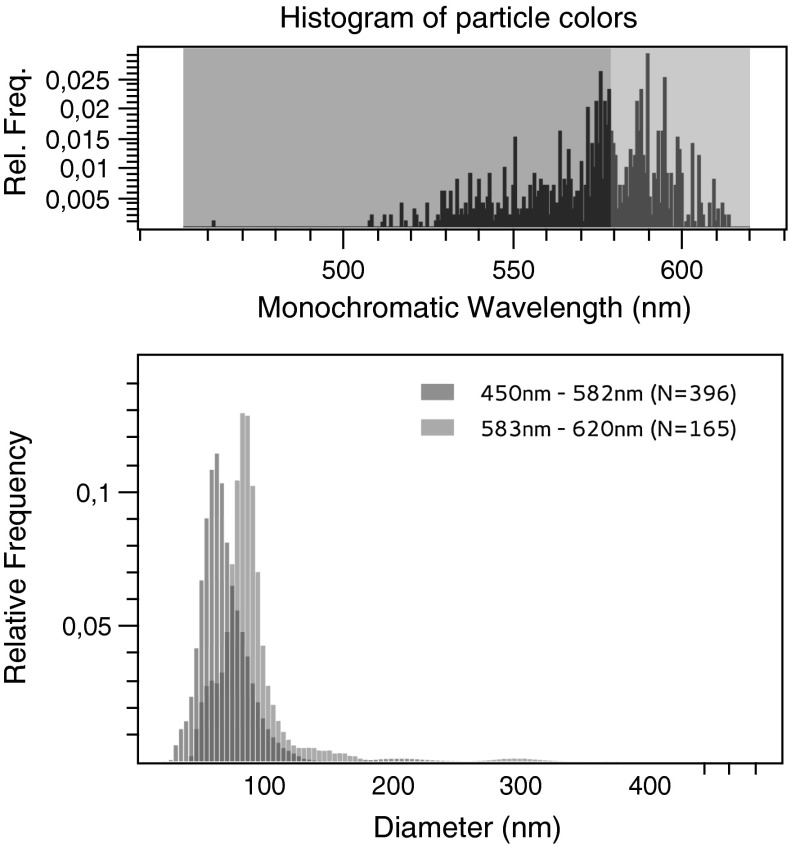
Assignment of color information to the size of gold NP. The size distribution from Au NPs shown in Fig. 3 was separated. Upper histogram: For the orange distribution, the wavelength range is 583–620 nm and the green size distribution includes particles with a range of 450–582 nm. The modal values are 60 and 80 nm, respectively. (Color figure online)

In the next step, we investigated how colors of Au NP elicited by dark field illumination can add to or even improve the determination of their size distribution. We found that using the SPR of Au NPs it was possible to identify the particle subpopulations in the bimodal Au NP suspension. Figure 3 shows a multi-colored set of diffusing particles with green and orange being the dominating colors. As outlined above, difference in plasmon resonance could reflect a different size, shape, or dielectric environment of the AuNP (Bingham and Willets 2009; Buecker 2007; El-Brolossy et al. 2008). As there was no change in color of each single particle over time, a torsion of an irregularly shaped particle was unlikely to account for this disparity. Furthermore, according to manufacturer’s specification, all visible Au NPs were designated as spherical. As NP was in the same chemical surrounding, divergent colors were most likely provoked by the different sizes. Figure 4 shows the size distribution estimated by NanoTrackJ. The modal value was 60 nm but the size distribution also exhibited a “shoulder” pointing to a 80 nm subpopulation of particles. A histogram was generated using the color information around the center of each particle which was transformed to the HSB color space. The hue was than mapped to its monochromatic wavelength. As the different colors of NP in Fig. 3 and also the histogram of particle colors (Fig. 4) suggested two different particle populations, we used the color thresholder inbuilt in NanoTrackJ and analyzed green-to-yellow particles (wavelength: 450–582 nm) and orange-to-red particles (wavelength: 583–620 nm) separately. This identified two different NP populations whose modal values exactly amounted to 60 and 80 nm, respectively (Fig. 5). It is furthermore noteworthy that these two populations were hardly separable without their color information. Also the cluster analysis method, previously devised by us to identify subpopulations of NP (Wagner et al. 2013) during NTA, was unable to separate the subpopulations of the current study.

### Detection limit of dark field nanoparticle tracking

The detection limit for NP viewed with DFM is hard to define, as it depends on the type of light source and its intensity, the microscope objective, and the amount of light passing the (variable) aperture. Also, the sensitivity of the camera is an important part of the experimental setup. With respect to the particles, the refractive index and the particular properties leading to plasmonic resonance are important to obtain a color image to be evaluated with NanoTrackJ. As stated by Boyer et al. (2002), the minimal detectable plasmonic particle size should be around 40 nm because the Rayleigh scattering decreases as the sixth power of diameter. In our experiments, 50 nm Au NP was visible by eye and could also be tracked with a color CCD or CMOS camera. In contrast, 50 nm polystyrene NP, which is easily tracked with the NanoSight System, could not be imaged with DFM as used here. In case that all optical elements were carefully adjusted images from diffusing 100 nm polystyrene beads became observable and could be tracked with sensitive cameras, suggesting a detection limit for non-plasmonic particles in the 100 nm range. Therefore, DFM will hardly lower the size limit of particles to be analyzed by tracking analysis, which is in the range of 10 nm for gold NPs. Instead, it is useful to analyze colored NP which, as shown here, can help to identify subpopulations of particles. First results have also shown that plasmonic particles undergo a color change secondary to cellular uptake (Wang et al. 2010). This opens new possibilities for visualizing alterations of the chemical surrounding, compartmentalization and/or corona formation of NPs, while simultaneously studying their movement inside cells.

## Conclusion

DFM is a valuable tool for characterizing nanoparticle suspensions. The technique is especially applicable for nanoparticle tracking from color videos of diffusing plasmonic NPs. The exploitation of the color information can improve the identification of particle subpopulations under defined conditions. This means that not only size information may be retrieved, as shown here, but also changes of chemical surrounding and/or particle coating may become experimentally accessible, e.g., during the uptake of NP by cells. Therefore, the open source software NanoTrackJ was devised as a tool for the expanding scientific community of nano researchers.

### Electronic supplementary material

Below is the link to the electronic supplementary material.

Supplementary material 1 (pdf 55 KB)

## Figures and Tables

**Fig. 6:**
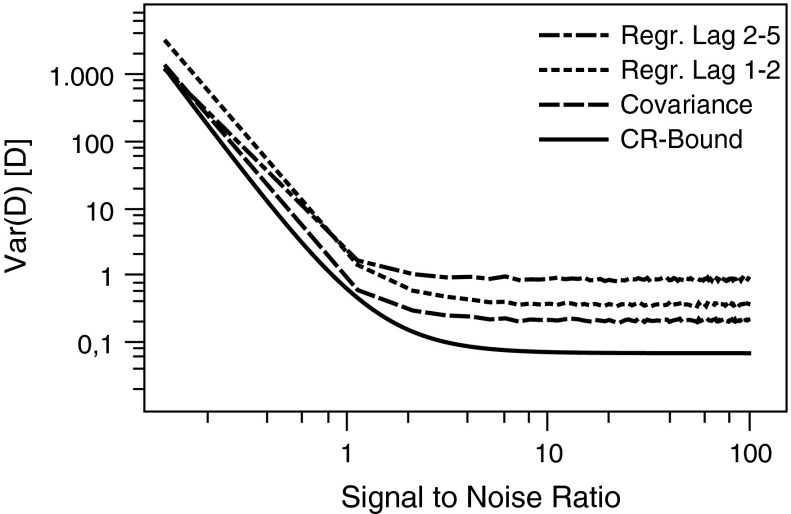
Variance for different diffusion coefficient estimators versus the signal to noise ratio for simulated trajectories

## Appendix

### Precision analysis: covariance estimator, regression estimator and the Cramer–Rao Bound

In this section, we will provide a comparison of the precision of the regression and covariance estimator under localization noise. Both estimators are accurate, which means that they are unbiased. The precision of an estimator is quantified by its variance. We will compare the variance of the estimators with each other and, secondly, also with the minimal attainable variance of an unbiased estimator, called the Cramer–Rao bound. To derive this bound, we need a model of the diffusion process which includes localization noise: Let $x[n]$ be the measured positions of a one dimensional diffusion of a particle with the diffusion coefficient $D$ then

10 $$\begin{aligned} x[n]&= x_{true}[n]+\xi _{n} \end{aligned}$$

11 $$\begin{aligned} x_{true}[n]&= x_{true}[n-1]+\beta _{n} \end{aligned},$$

where $\beta$ is normal random variable $N(\mu _{\beta }=0,\sigma _{\beta }^{2}=2D\triangle t)$ which represents the step length distribution, $\triangle t$ is the time between two measurements, $x_{true}[n]$ is the true position of the particle, and $\xi$ is the normal distributed localization noise with mean $\mu _{\xi }=0$ and standard deviation $\sigma _{\xi }$. For the expected value and the variance of the position differences apply

12 $$\begin{aligned} \left\langle x[n]-x[n-1]\right\rangle&= \left\langle \beta _{n}\right\rangle +\left\langle \xi {}_{n}\right\rangle +\left\langle -\xi {}_{n-1}\right\rangle =0 \end{aligned}$$

13 $$\begin{aligned} \left\langle (x[n]-x[n-1])^{2}\right\rangle&= \left\langle \left( \beta _{n}+\xi _{n}-\xi _{n-1}\right) ^{2}\right\rangle \end{aligned}$$

14 $$\begin{aligned}&= 2D\triangle t+2\sigma _{\xi }^{2} \end{aligned}$$

Now we are able to define the likelihood function of the position differences. Let $x=(x[0],...,x[N-1])$ then the likelihood is

15 $$\begin{aligned} p_{\chi }(x;D)&= \prod \limits _{1}^{N-1}\frac{\exp \left( -\frac{(x[n]-x[n-1])^{2}}{4D\triangle t+4\sigma _{\xi }^{2}}\right) }{\sqrt{4\pi D\triangle t+4\pi \sigma _{\xi }^{2}}}\end{aligned}$$

16 $$\begin{aligned} \,&= \frac{\exp \left( -\frac{S}{4D\triangle t+4\sigma _{\xi }^{2}}\right) }{(4\pi D\triangle t+4\pi \sigma _{\xi }^{2})^{\frac{(N-1)}{2}}} \end{aligned},$$

where

17 $$\begin{aligned} S=\sum \limits _{n=1}^{n=N-1}(x[n]-x[n-1])^{2} \end{aligned}$$

The second derivative of the log-likelihood is

18 $$\begin{aligned} \frac{\partial ^{2}\, ln\, p(x;D)}{\partial ^{2}D}&= \frac{(N-1)\triangle t{}^{2}}{2(D\triangle t+\sigma _{\xi }^{2})^{2}}-\frac{\triangle t{}^{2}S}{2(D\triangle t+\sigma _{\xi }^{2})^{3}} \end{aligned}$$

and taking the expectation results in

19 $$\begin{aligned} \left\langle \frac{\partial ^{2}\, ln\, p(x;D)}{\partial ^{2}D}\right\rangle&= -\frac{(N-1)\triangle t{}^{2}}{2(D\triangle t+\sigma _{\xi }^{2})^{2}} \end{aligned}$$

This leads to the Cramer–Rao inequality

20 $$\begin{aligned} var(\hat{D)}\ge -\frac{1}{\left\langle \frac{\partial ^{2}\, ln\, p(x;D)}{\partial ^{2}D}\right\rangle }=\frac{2(D\triangle t+\sigma _{\xi }^{2})^{2}}{(\triangle t)^{2}(N-1)} \end{aligned}$$

To compare the precision of the estimators, we have carried out Monte Carlo simulations. Therefore, we define a signal to noise ratio as

21 $$\begin{aligned} SNR=\frac{\sqrt{2D\triangle t}}{\sigma _{\xi }} \end{aligned}$$

For each signal to noise ratio, 3,000 trajectories with tracklength of 30 were generated and the variance was calculated. Figure 6 shows the precision comparison of the covariance estimator suggested by Vestergaard (2012) and the regression estimators based on the time lag $2-5$ ($R_{2-5}$) and $1-2$ ($R_{1-2}$) as suggested by Ernst and Köhler (2013) and Wieser and Schütz (2008), respectively.

For signal to noise ratios below 1, the $R_{2-5}$ estimator has a higher precision than the $R_{1-2}$ estimator. For more practically relevant signal to noise ratios (>1), the $R_{1-2}$ estimator has a significant higher precision. The covariance estimator provides a higher precision as both regression estimators for all signal to noise ratios but does not attain the Cramer–Rao bound even for large SNRs. However, the Cramer–Rao bound does not provide any information if such an estimator even exists. We, therefore, recommend that the covariance estimator should be used instead of the regression estimator because of its higher precision and easy implementation which needs no numerical fitting.
