# Supplementary material for: Dark field nanoparticle tracking analysis for size characterization of plasmonic and non-plasmonic particles
Source: J Nanopart Res. 2014 May 1;16(5):2419. doi: 10.1007/s11051-014-2419-x (PMC4021069; doi:10.1007/s11051-014-2419-x)
Supplement: Supplementary file 1 — Supplementary material 1 (pdf 55 KB) [file 11051_2014_2419_MOESM1_ESM.pdf]

Supplementary Information for
„Dark field nanoparticle tracking analysis for size characterization of plasmonic and non plasmonic particles“

0. General

This document contains some information helpful to reproduce the measurements. The tables in section 2 specify all software settings used for the measurements. The references to the analyzed video files are listed in the table row “Source”. The video files are online available and under a creative commons licence. To reproduce the measurements you have to install Fiji/ImageJ and also the NanoTrackJ plugin. The version used in this paper is avaible under:

NanoTrackJ: Size characterization of freely diffusing nanoparticles by nanoparticle tracking. Thorsten Wagner, Martin Wiemann, Hans-Gerd Lipinski. figshare.
http://dx.doi.org/10.6084/m9.figshare.805052

1. Naming convention

All files are named according to the following convention:

Type_T-X_C-X_FPS-X
with

- Type
  - DF = Dark Field (Cytoviva Condensor on an Olympus BX51 microscope)
  - NS = NanoSight LM 10 (green laser)
- P-X = Particle
  - P-Au: Gold
  - P-Ps: Polystyrol
- S-X = Nominal particle size in nm
- T-X = Temperatur in C°
- C-X = Camera Type
  - C-A: Andor-DL-658-OEM
  - C-E: PCO Pixelfly Edge
  - C-O: Canon EOS 5D Mark II
- FPS-X = Frames per Second

2. Software settings to reproduce the results

Figure 1A:

Software	Nanosight NTA 2.3
Video file	NS_P-Ps_S-100_T-22_C-A_FPS-30.avi (first 1800 frames)
Source	Wagner, Thorsten; Wiemann, Martin; Lipinski, Hans-Gerd (2013): Video recording of freely diffusing 100nm polystyrene nanoparticles using nanoparticle tracking analysis. figshare. http://dx.doi.org/10.6084/m9.figshare.879651

The settings used for this plot can be found in the supplementary files named as the video on the Journal website.

Figure 1B:

Software	NanoTrackJ
Video file	NS_P-Ps_S-100_T-22_C-A_FPS-30.avi (first 1800 frames)
Source	Wagner, Thorsten; Wiemann, Martin; Lipinski, Hans-Gerd (2013): Video recording of freely diffusing 100nm polystyrene nanoparticles using nanoparticle tracking analysis. figshare. http://dx.doi.org/10.6084/m9.figshare.879651
Center estimator	Maxima
Diffusion coefficient estimator	Covariance
Min. expected particle size	90 nm
Searching radius	13.34 px
Min. number of steps per track	20
Temperature	22,5 °C
Pixel size	164 nm
Frame rate	30 FPS
Linear drift corrected	True
Walker's method used:	True
Walker's method min size:	800nm
Mean size (Maxima Dialog)	3
Tolerance (Maxima Dialog)	15

Figure 1C:

Software	Nanosight NTA 2.3
Video file	NS_P-Ps_S-200_T-24_C-A_FPS-30.avi
Source	Wagner, Thorsten; Wiemann, Martin; Lipinski, Hans-Gerd (2013): Video recording of freely diffusing 200nm polystyrene nanoparticles using nanoparticle tracking analysis. figshare. http://dx.doi.org/10.6084/m9.figshare.879662

The settings used for this plot can be found in the supplementary files named as the video on the Journal website.

Figure 1D:

Software	NanoTrackJ
Video file	NS_P-Ps_S-200_T-24_C-A_FPS-30.avi
Source	Wagner, Thorsten; Wiemann, Martin; Lipinski, Hans-Gerd (2013): Video recording of freely diffusing 200nm polystyrene nanoparticles using nanoparticle tracking analysis. figshare. http://dx.doi.org/10.6084/m9.figshare.879662
Center estimator	Maxima
Diffusion coefficient estimator	Covariance

Min. expected particle size	190nm
Searching radius	9.37 px
Min. number of steps per track	20
Temperature	24.05 °C
Pixel size	164 nm
Frame rate	30 FPS
Linear drift corrected	True
Walker's method used:	True
Walker's method min size:	800 nm
Mean size (Maxima Dialog)	4
Tolerance (Maxima Dialog)	27

Figure 2 (left):

Software	NanoTrackJ
Video file	DF_P-Ps_S-100_T-24_C-E_FPS-30.avi
Source	Wagner, Thorsten; Wiemann, Martin; Lipinski, Hans-Gerd (2013): Video recording of freely diffusing 100nm polystyrene nanoparticles using dark field microscopy. figshare. http://dx.doi.org/10.6084/m9.figshare.878145
Center estimator	Maxima
Diffusion coefficient estimator	Covariance
Min. expected particle size	90nm
Searching radius	35.44 px
Min. number of steps per track	20
Temperature	24 °C
Pixel size	63 nm
Frame rate	30 FPS
Linear drift corrected	True
Walker's method used:	True
Walker's method min size:	800 nm
Mean size (Maxima Dialog)	1
Tolerance (Maxima Dialog)	5

Figure 2 (right):

Software	NanoTrackJ
Video file	DF_P-Ps_S-200_T-25_C-E_FPS-30.avi
Source	Video recording of freely diffusing 200nm polystyrene nanoparticles using dark field microscopy. Thorsten Wagner, Martin Wiemann, Hans-Gerd

	Lipinski. Figshare. http://dx.doi.org/10.6084/m9.figshare.878107
Center estimator	Maxima
Diffusion coefficient estimator	Covariance
Min. expected particle size	190 nm
Searching radius	24.72 px
Min. number of steps per track	20
Temperature	25 °C
Pixel size	63 nm
Frame rate	30 FPS
Linear drift corrected	True
Walker's method used:	True
Walker's method min size:	800 nm
Mean size (Maxima Dialog)	1
Tolerance (Maxima Dialog)	9

Figure 4:

Software	NanoTrackJ
Video file	DF_P-Au_S-60_T-24_C-O_FPS-25.tif
Source	Wagner, Thorsten; Wiemann, Martin; Lipinski, Hans-Gerd (2013): Video recording of freely diffusing 60 nm and 80 nm gold nanoparticles using dark field microscopy. figshare. http://dx.doi.org/10.6084/m9.figshare.879664
Center estimator	Maxima
Diffusion coefficient estimator	Covariance
Min. expected particle size	50 nm
Searching radius	18.03 px
Min. number of steps per track	20
Temperature	24 °C
Pixel size	182 nm
Frame rate	25 FPS
Linear drift corrected	True
Walker's method used:	True
Walker's method min size:	800 nm
Mean size (Maxima Dialog)	4
Tolerance (Maxima Dialog)	12

Figure 5:
Same configruation as used for figure 4. The orange colored distribution was measured using the color threshold with an upper hue of 60 and a lower hue of 0.
The green colored distribution was measured using the color threshold with an upper hue of 285 and a lower hue of 60.
